# Association between exposure to multiple air pollutants, transportation noise and cause-specific mortality in adults in Switzerland

**DOI:** 10.1186/s12940-023-00983-y

**Published:** 2023-03-27

**Authors:** Danielle Vienneau, Massimo Stafoggia, Sophia Rodopoulou, Jie Chen, Richard W. Atkinson, Mariska Bauwelinck, Jochem O. Klompmaker, Bente Oftedal, Zorana J. Andersen, Nicole A. H. Janssen, Rina So, Youn-Hee Lim, Benjamin Flückiger, Regina Ducret-Stich, Martin Röösli, Nicole Probst-Hensch, Nino Künzli, Maciek Strak, Evangelia Samoli, Kees de Hoogh, Bert Brunekreef, Gerard Hoek

**Affiliations:** 1 Department of Epidemiology and Public Health, Swiss Tropical and Public Health Institute, Kreuzstrasse 2, Allschwil, CH-4123 Switzerland; 2 University of Basel, Basel, Switzerland; 3 Department of Epidemiology, Lazio Region Health Service / ASL Roma 1, Rome, Italy; 4 Institute of Environmental Medicine, Karolinska Institutet, Stockholm, Sweden; 5 Department of Hygiene, Epidemiology and Medical Statistics, Medical School, National and Kapodistrian University of Athens, Athens, Greece; 6 Institute for Risk Assessment Sciences, Utrecht University, Utrecht, Netherlands; 7 Population Health Research Institute, St George’s, University of London, London, UK; 8 Interface Demography – Department of Sociology, Vrije Universiteit Brussel, Pleinlaan 2, Brussels, 1050 Belgium; 9 National Institute for Public Health and the Environment (RIVM), Bilthoven, Netherlands; 10 Department of Environmental Health, Norwegian Institute of Public Health, Oslo, Norway; 11 Section of Environmental Health, Department of Public Health, University of Copenhagen, Copenhagen, 1165 Denmark

**Keywords:** Traffic-related pollution, Air, Noise, Metals, Mortality, Cardiovascular, Respiratory

## Abstract

**Background:**

Long-term exposure to air pollution and noise is detrimental to health; but studies that evaluated both remain limited. This study explores associations with natural and cause-specific mortality for a range of air pollutants and transportation noise.

**Methods:**

Over 4 million adults in Switzerland were followed from 2000 to 2014. Exposure to PM_2.5_, PM_2.5_ components (Cu, Fe, S and Zn), NO_2_, black carbon (BC) and ozone (O_3_) from European models, and transportation noise from source-specific Swiss models, were assigned at baseline home addresses. Cox proportional hazards models, adjusted for individual and area-level covariates, were used to evaluate associations with each exposure and death from natural, cardiovascular (CVD) or non-malignant respiratory disease. Analyses included single and two exposure models, and subset analysis to study lower exposure ranges.

**Results:**

During follow-up, 661,534 individuals died of natural causes (36.6% CVD, 6.6% respiratory). All exposures including the PM_2.5_ components were associated with natural mortality, with hazard ratios (95% confidence intervals) of 1.026 (1.015, 1.038) per 5 µg/m^3^ PM_2.5_, 1.050 (1.041, 1.059) per 10 µg/m^3^ NO_2_, 1.057 (1.048, 1.067) per 0.5 × 10^–5^/m BC and 1.045 (1.040, 1.049) per 10 dB Lden total transportation noise. NO_2_, BC, Cu, Fe and noise were consistently associated with CVD and respiratory mortality, whereas PM_2.5_ was only associated with CVD mortality. Natural mortality associations persisted < 20 µg/m^3^ for PM_2.5_ and NO_2_, < 1.5 10^–5^/m BC and < 53 dB Lden total transportation noise. The O_3_ association was inverse for all outcomes. Including noise attenuated all outcome associations, though many remained significant. Across outcomes, noise was robust to adjustment to air pollutants (e.g. natural mortality 1.037 (1.033, 1.042) per 10 dB Lden total transportation noise, after including BC).

**Conclusion:**

Long-term exposure to air pollution and transportation noise in Switzerland contribute to premature mortality. Considering co-exposures revealed the importance of local traffic-related pollutants such as NO_2_, BC and transportation noise.

**Supplementary Information:**

The online version contains supplementary material available at 10.1186/s12940-023-00983-y.

## Introduction

Air pollution is an important contributor to morbidity and mortality, with an estimated 6.7 million deaths due to long-term exposure to ambient particulate air pollution worldwide [1]. Though air pollution levels in Europe and premature deaths attributed to particulate matter < 2.5 µm (PM_2.5_) have generally declined since the mid 2000’s, the burden of disease remains high [2]. The much reduced recommendations for low limit values in the 2021 WHO air quality guidelines (AQG) [3], and the growing body of evidence from developed nations, clearly signal that even low levels of air pollution are harmful [4–9]. Likewise exposure to noise from transportation sources is also known to be detrimental to health and linked to mortality [10–15], with the most recent health policy instrument being the WHO Environmental Noise Guidelines (ENG) for the European Region [16]. Though studies with similar spatially resolved air pollution and noise exposure data are limited, some suggest the effects of noise are independent [12, 17–19] thus leading to additional health burden from diseases also associated with air pollution. For example, a comparative health risk assessment for Switzerland using data from 2010 indicated the external costs of the transportation noise burden is equal to that of air pollution [20].

The multicenter Effects of Low-Level Air Pollution: A Study in Europe (ELAPSE) study investigated associations of long-term exposure to air pollution with natural and cause-specific mortality in both a pooled analysis of over 300,000 adults from eight population-based cohorts with detailed confounder data [21], and a meta-analysis of seven administrative cohorts—including the Swiss National Cohort (SNC)—for a total of over 28 million adults [22]. In both analyses, PM_2.5_, nitrogen dioxide (NO_2_) and black carbon (BC) were clearly associated with increased risk of natural, cardiovascular (CVD) and non-malignant respiratory mortality. These associations persisted in subsets of the population residing in areas with low concentrations; and for CVD, the outcome for which noise was considered, the air pollution associations were generally robust to adjustment for transportation noise. In six of the administrative cohorts including the SNC [23], and the pooled cohorts in ELAPSE [24], further investigation of eight PM_2.5_ components (including copper (Cu), iron (Fe), sulphur (S) and zinc (Zn)) showed associations with natural mortality for most components that often attenuated after considering PM_2.5_ mass. All air pollution concentration estimates were derived from harmonized, Europe-wide models [25, 26]. Though transportation noise was considered in some administrative cohorts of ELAPSE as a further adjustment for CVD, it was given less attention because the exposure models were cohort specific and thus heterogeneous. Since publication of the WHO ENG [16], newer studies on other outcomes have suggested noise may also be related to respiratory disease and natural mortality [11, 14, 17].

The often high correlations between exposures deriving from similar sources make it difficult to discern which exposure, or combination thereof, drive the mortality associations within any given jurisdiction. The spatial variation and levels of some exposures, such as PM_2.5_ components and BC, further depend on the local context in which other co-exposures related to traffic, such as transportation noise, may also play a role. Furthermore, air pollutant concentration maps are often estimated via land-use regression approaches, potentially increasing their correlations when similar predictors are used for different pollutants. The complexity of the total exposure environment for disentangling health effects imposes clear challenges to the downstream regulation and public health decision making. Insight into the country-specific mortality associations is thus crucial for the authorities engaged in setting guidelines and standards for air quality and noise, given that the Environmental Law requires protection of all people from emissions that harm health or impair wellbeing [27]. Extending the analyses in ELAPSE to more thoroughly study transportation noise as a co-exposure, the aim of this study was to investigate the associations between long-term exposure to air pollution and total transportation noise with natural, CVD and respiratory mortality in the SNC. To assess the independent effects of air pollution and transportation noise, particular emphasis was on specification of two exposure models. An additional aim was to gain insight into the associations at very low levels via subset analyses. Exposures included PM_2.5_, NO_2_, BC and warm season O_3_ (subsequently referred to as O_3_ warm), several PM_2.5_ components (Cu, Fe, S and Zn) and transportation noise.

## Methods

### Study population

The Swiss National Cohort (SNC) is an administrative cohort that links the decennial national census (for 1990 and 2000) and Registry Based Census (from 2010 onward) with births, mortality and emigration [28, 29]. With compulsory participation in the census, virtually all residents in Switzerland are represented, i.e. 98.6% in the 04 December 2000 census [30]. The SNC was approved by the Ethics Committees of the Cantons of Zurich and Bern.

Treated as a closed cohort, this study used data from 04 December 2000 (i.e. baseline date) to 31 December 2014 (i.e. end of follow-up). In total, 7.28 million observations of all ages were available at baseline. With the focus on mortality outcomes in adults, the analysis included 4.19 million individuals after excluding the following observations: individuals below 30 years of age (*n* = 2.6 million) and those with missing residential coordinates or designated as living in an institution (*n* = 0.4 million). A further 0.1 million with missing individual characteristics, specifically education and socio-economic position (SEP), were excluded to ensure a study population with complete data for use in all analyses (Supplement Table S1).

### Outcome definition

The outcomes under investigation were primary causes of death from natural causes (International Classification of Disease version 10 [ICD-10]: A00 – R99), CVD (ICD-10: I10 – I70), and non-malignant respiratory disease (ICD-10: J00 – J99).

### Exposure assessment

Annual average ambient PM_2.5_, NO_2_ and BC concentrations, in addition to O_3_ warm (based on maximum 8-h running means, during April to September), were available from the European 100 × 100 m hybrid land use regression models for 2010 developed within ELAPSE [26]. Exposure to PM_2.5_ components including the four investigated here (Cu, Fe and Zn as indicators of non-tailpipe emissions, and S representing long-range transported inorganic aerosols) were also developed within ELAPSE [25]; models derived with supervised linear regression were used. Five-fold hold-out validation was used to evaluate the models, with *R*^2^: 0.66 for PM_2.5_; 0.58 for NO_2_; 0.51 for BC; 0.60 for O_3_ warm; 0.48 for Cu; 0.48 for Fe; 0.41 for Zn and 0.79 for S [25, 26]. These models for 2010 were a priori defined as the main exposure models in ELAPSE, and were assigned to participant baseline addresses.

Transportation noise exposure for Switzerland derived from the SiRENE project (Short and Long Term Effects of Transportation Noise Exposure) [31, 32], in which noise was modelled by source at each dwelling façade (i.e. by floor of residence) at decennial census years. Models for road traffic, railways and aircraft were respectively based on the sonRoad (with StL-86 propagation model), sonRail (with SEMIBEL propagation model) and FLULA2. The Lden metric (i.e. weighted energetic average of Leq,day (07:00–19:00), Leq,evening (19:00–23:00) and Leq,night (23:00–07:00) with a respective penalty of 5 and 10 dB applied to the evening and night) was computed for each noise source. The energetic sum of these three sources was then determined to derive total transportation noise for each dwelling [33], based on the noise level at the maximum exposed façade. This was the a priori main noise exposure; road traffic noise was included for comparison. Noise exposure from the year 2001 was used to align with the cohort baseline. Noise exposure is stable over time, with moderate to high correlations (r 0.67–0.96 depending on source) previously reported for the SNC [15].

### Covariates

Individual-level characteristics included sex (female/male), marital status (single, married, widowed, divorced), education level (compulsory education or less, upper secondary level education, tertiary level education), mother tongue (German and Rhaeto-Romansch, French, Italian, other language), and nationality (Swiss, non-Swiss). Area-level SEP variables were also developed to provide broader area context not captured by the individual covariates. These were calculated by aggregating the relevant individual variables, including the Swiss-SEP index of socio-economic position (i.e. calculated for small local areas of 50 nearest neighbours as described in Panczak et al. [34]). The following were calculated at “neighbourhood” (*n* = 3,175 postcode areas) and “regional” (*n* = 26 Swiss cantons) level: composite score (mean of the Swiss-SEP index); unemployment rate (% working age population [20 to 65 years] unemployed); low education rate (% adults with compulsory or less education); and high education rate (% adults with tertiary education or higher). All covariates were available for the baseline year.

### Statistical analysis

Associations were analysed using the Cox proportional hazards model, with age as the underlying time scale. Models were stratified by sex, and clustered by neighbourhood to properly adjust the standard errors of the estimates for the correlations of subjects residing in the same neighbourhood [22, 35]. Participants were followed until the event, emigration, death by another cause or end of follow-up, which ever came first.

The adjustment strategy followed the ELAPSE study protocol. Model 1 included only age (time axis), sex (as strata) and neighbourhood (as cluster). Model 2 added individual level variables applicable to the Swiss context, specifically: education level, occupational status, marital status, country of origin, and mother tongue. Model 3, the a priori main model, added the four types of area-level SEP variables described above, at both the neighbourhood and regional level. The two exposure models were based on Model 3, and included a further adjustment for one of the following main exposures, in turn: PM_2.5_, NO_2_, BC, O_3_ warm or total transportation noise. Hazard ratios were computed including linear terms for the exposure(s) and were expressed per standard units (PM_2.5_ per 5 µg/m^3^, NO_2_ per 10 µg/m^3^, BC per 0.5 × 10^–5^/m, O_3_ warm per 10 µg/m^3^; PM_2.5_ Cu per 5 ng/m^3^, PM_2.5_ Fe per 100 ng/m^3^, PM_2.5_ S per 200 ng/m^3^, PM_2.5_ Zn per 10 ng/m^3^; and total transportation noise or road traffic noise exposure per 10 dB Lden). Previous analyses showed linear to supralinear associations in this cohort [15, 22], thus linear exposure terms were considered justified.

To look deeper into the associations at very low levels of the main exposures, subsets were defined by removing participants residing in areas above pre-specified values including guideline limit values. Thus using main Model 3, associations below the following levels were investigated: PM_2.5_ below 25 (EU limit value), 20, 15 and 12 μg/m^3^; NO_2_ below 40 (EU limit value; former 2005 WHO air quality guideline value), 30 and 20 μg/m^3^; BC below 3.0, 2.5, 2.0 and 1.5 10^–5^/m and O_3_ warm below 120 and 100 μg/m^3^. There were an insufficient number of exposed individuals to investigate associations for PM_2.5_ and NO_2_ below 5 and 10 μg/m^3^ (2021 WHO air quality guideline values). Associations below 60, 55 and 53 dB Lden total transportation noise, with the latter being the WHO ENG level for road traffic noise [16], were also investigated. Additional analyses based on two exposure models included investigating effect modification by sex as well as a non-movers analysis. All analyses were conducted in R v3.4.0 using common scripts developed in ELAPSE.

## Results

### Study description

In total 4,188,175 adults over the age of 30 years on 04 December 2000 with complete exposure and covariate data were included in the cohort (Table S1). The cohort included slightly more women than men (52.0%), with a high proportion of Swiss nationals (83.1%). The majority had a mother tongue of German or Rhaeto-Romansch (65.1%), were employed (61.4%), and married (69.3%) at baseline. During follow up (mean of 12.7 years, and 53,344,296 person years), 661,534 deaths due to natural causes were recorded, of which 36.6% and 6.6% were due to CVD and respiratory mortality, respectively. The mean age of death was over 70 years for all investigated causes. Compared to the study population, a notably higher proportion of deaths were in those who were widowed or had low education (Table 1).

**Table 1:** Characteristics of the study population and deaths during follow-up

**Variable**	**Study population (SNC)**	**Deaths during follow-up**		
		**Natural cause**	**Cardiovascular**	**Respiratory**
N participants total	4,293,521	673,946	246,840	44,489
**N participants with complete data**	**4,188,175**	**661,534**	**241,985**	**43,612**
N person years	53,344,296			
**Individual level covariates**				
Age (mean +–Sd)	52.7 (15.2)	72.1 (12.3)	75.7 (10.9)	74.6 (10.6)
Women (%)	52.0	50.4	52.9	44.7
Country origin (% Swiss)	83.1	92.4	94.1	92.8
Marital status (%)				
Single	14.0	9.6	9.2	9.7
Married	69.3	55.7	51.1	54.0
Divorced	8.7	7.8	6.4	8.3
Widowed	8.1	26.8	33.3	27.9
Education (%)				
Low (compulsory education or less)	24.5	40.4	44.6	43.8
Medium (upper secondary level)	52.7	46.5	44.0	45.0
High (tertiary level)	22.7	13.2	11.3	11.2
Occupational status (%)				
Employed/self-employed	61.4	18.1	11.9	11.5
Unemployed	2.2	1.1	0.7	0.9
Homemaker	14.6	8.3	4.8	7.3
Retired	21.8	72.4	82.6	80.4
Mother tongue (%)				
German and Rhaeto-Romansch	65.1	69.8	73.7	65.6
French	19.6	20.9	18.5	24.6
Italian	7.4	6.9	6.1	7.8
Other	8.0	2.3	1.7	2.0
**Area level covariates** (mean +–Sd)				
Neighborhood socio-economic position (SEP) score	63.0 (7.3)	62.7 (7.3)	62.6 (7.3)	62.1 (7.4)
Neighborhood unemployment rate	3.5 (1.5)	3.5 (1.6)	3.5 (1.5)	3.6 (1.6)
Neighborhood low education rate	28.4 (7.3)	28.9 (7.4)	28.9 (7.4)	29.4 (7.5)
Neighborhood high education rate	19.8 (7.5)	19.6 (7.5)	19.2 (7.3)	19.4 (7.6)
**Exposures** (mean +–Sd)				
PM_2.5_ (µg/m^3^)	15.9 (2.4)	16.0 (2.4)	15.9 (2.4)	15.9 (2.6)
NO_2_ (µg/m^3^)	23.7 (7.4)	24.0 (7.6)	23.6 (7.5)	24.0 (7.9)
BC (10^–5^/m)	1.67 (0.35)	1.69 (0.36)	1.67 (0.35)	1.69 (0.40)
O_3_ warm (µg/m^3^)	94.8 (5.9)	94. 7 (6.1)	94.9 (6.0)	94.8 (6.3)
PM_2.5_ Cu (ng/m^3^)	5.2 (2.7)	5.3 (2.7)	5.1 (2.7)	5.2 (2.9)
PM_2.5_ Fe (ng/m^3^)	108.4 (46.9)	110.3 (48.0)	107.9 (46.7)	110.1 (49.7)
PM_2.5_ S (ng/m^3^)	646.6 (85.0)	646.9 (87.8)	644.1 (87.4)	644.0 (92.3)
PM_2.5_ Zn (ng/m^3^)	20.8 (18.5)	21.4 (19.0)	21.0 (19.1)	21.0 (18.9)
Total transportation noise (dB Lden)	55.9 (8.2)	56.4 (8.2)	56.3 (8.2)	56.6 (8.3)
Road traffic noise (dB Lden)	54.2 (8.1)	54.8 (8.1)	54.8 (8.2)	55.0 (8.2)

Mean (standard deviation, SD) exposures were 15.9 µg/m^3^ (2.4) for PM_2.5_, 23.7 µg/m^3^ (7.4) for NO_2_, 1.67 10^–5^/m (0.35) for BC and 94.8 µg/m^3^ (5.9) for O_3_ warm. As indicated by the small standard deviation, the contrast for O_3_ warm was small. The most abundant PM_2.5_ components were S (646.6 [85.0] ng/m^3^) followed by Fe (108.4 [46.9] ng/m^3^) (Table 1, Table S2). Spearman correlations amongst the air pollutant exposures were moderate to high. For the main pollutants the highest correlation was between NO_2_ and BC (0.91), and both were correlated > 0.7 with PM_2.5_. O_3_ warm was negatively correlated with all exposures, the highest being NO_2_, BC and PM_2.5_ (< -0.65). Cu and Fe were highly correlated with NO_2_ and BC (> 0.88). Amongst PM_2.5_ components, Cu and Fe were almost perfectly correlated (0.97). The mean (SD) total transportation noise and road traffic noise exposures were 55.9 (8.2) and 54.2 (8.1) dB Lden, respectively. Total transportation noise was dominated by road traffic noise, with a correlation of 0.89 in this study population (Table S3). In terms of correlations, noise was low to moderately correlated (within ± 0.40) with each air pollutant including the PM_2.5_ components. Most exposures also showed a low positive correlation with neighbourhood SEP score, indicating higher exposures in high SEP neighbourhoods (with these in urban areas in Switzerland). Exceptions were O_3_ warm in the opposite direction (low negative) and noise which were uncorrelated (Table S3). The corresponding mean exposures by quintiles of neighbourhood SEP score showed a consistent pattern of higher air pollution (lower for O_3_ warm) with higher SEP across the whole population, and within urban and rural populations (Table S4).

### Single and two exposure models

For most air pollution exposures the hazard ratios became stronger with increasing covariate adjustment from Model 1 to main Model 3. The opposite patterns were found for O_3_ warm (i.e. stronger inverse associations with increasing adjustment). A clear pattern in covariate adjustment for total transportation noise was less obvious (Table S5).

All exposures were associated with natural mortality in single exposure models, including O_3_ warm though in the inverse direction (Table 2). The hazard ratios were 1.026 (1.015, 1.038) per 5 µg/m^3^ PM_2.5_, 1.050 (1.041, 1.059) per 10 µg/m^3^ NO_2_, 1.057 (1.048, 1.067) per 0.5 × 10^–5^/m BC, 0.946 (0.939, 0.954) per 10 µg/m^3^ O_3_ warm and 1.045 (1.040, 1.049) per 10 dB Lden total transportation noise (road traffic noise as exposure gave highly similar results). PM_2.5_ components (Cu, Fe, S and Zn) were also significantly associated with natural mortality in single exposure models. PM_2.5_ (mass and the individual components) associations attenuated, often to the null, after adjusting for NO_2_, BC or O_3_ warm; whereas including PM_2.5_ did not change the other single air pollutant exposure associations. O_3_ warm associations slightly attenuated when noise was introduced. NO_2_ and BC associations only slightly decreased though remained indicative of an association in models including noise, with slightly more attenuation when adjusting for road traffic vs. total transportation noise. The noise associations were robust to air pollution co-exposure adjustment; for example, the natural mortality association after BC adjustment was 1.037 (1.033, 1.042) per 10 dB Lden total transportation noise. HRs for noise were less affected by air pollution adjustment than air pollution HRs were for noise adjustment.

**Table 2:** Single vs. two exposure model hazard ratios (95% confidence intervals) for associations with mortality by cause (Model 3)

**Outcome**	**Exposure**	**Increment**	**Single**	**Adjusted for PM**_**2.5**_	**Adjusted for NO**_**2**_	**Adjusted for BC**	**Adjusted for O**_**3**_** warm**	**Adjusted for total noise**	**Adjusted for road traffic noise**
Natural cause mortality	PM_2.5_	5 µg/m^3^	1.026 (1.015, 1.038)	-	0.992 (0.978, 1.005)	0.989 0.976, 1.002)	1.000 (0.989, 1.012)	1.012 (1.001, 1.023)	1.013 (1.001, 1.024)
	NO_2_	10 µg/m^3^	1.050 (1.041, 1.059)	1.053 (1.043, 1.063)	-	1.017 (1.003, 1.032)	1.033 (1.023, 1.043)	1.029 (1.021, 1.038)	1.026 (1.017, 1.035)
	BC	0.5 × 10^–5^/m	1.057 (1.048, 1.067)	1.062 (1.051, 1.072)	1.041 (1.026, 1.056)	-	1.040 (1.029, 1.051)	1.035 (1.026, 1.044)	1.031 (1.022, 1.041)
	O_3_ warm	10 µg/m^3^	0.946 (0.939, 0.954)	0.946 (0.938, 0.955)	0.965 (0.955, 0.975)	0.969 (0.959, 0.978)	-	0.962 (0.955, 0.970)	0.963 (0.956, 0.971)
	PM_2.5_ Cu	5 ng/m^3^	1.067 (1.054, 1.080)	1.064 (1.050, 1.078)	1.014 (0.996, 1.033)	0.993 (0.973, 1.014)	1.037 (1.022,1.051)	1.040 (1.028,1.053)	1.035 (1.023, 1.048)
	PM_2.5_ Fe	100 ng/m^3^	1.085 (1.070, 1.100)	1.082 (1.066, 1.098)	1.041 (1.018, 1.064)	1.020 (0.994, 1.047)	1.056 (1.039,1.072)	1.052 (1.038,1.067)	1.046 (1.032, 1.061)
	PM_2.5_ S	200 ng/m^3^	1.035 (1.020, 1.051)	1.027 (1.009, 1.045)	0.989 (0.972, 1.007)	0.989 (0.973, 1.005)	0.995 (0.980,1.011)	1.014 (1.000,1.028)	1.012 (0.998, 1.026)
	PM_2.5_ Zn	10 ng/m^3^	1.004 (1.002, 1.006)	1.003 (1.001, 1.005)	1.001 (0.999, 1.003)	1.000 (0.998, 1.002)	1.003 (1.001,1.005)	1.003 (1.001,1.005)	1.003 (1.001, 1.005)
	Total Noise	10 dB Lden	1.045 (1.040, 1.049)	1.044 (1.039, 1.048)	1.038 (1.034, 1.043)	1.037 (1.033, 1.042)	1.039 (1.035, 1.044)	-	-
	Road traffic Noise	10 dB Lden	1.046 (1.042, 1.050)	1.045 (1.041, 1.050)	1.040 (1.035, 1.044)	1.039 (1.034, 1.043)	1.041 (1.037, 1.045)	-	-
CVD mortality	PM_2.5_	5 µg/m^3^	1.026 (1.008, 1.044)	-	1.011 (0.991, 1.032)	1.005 (0.986, 1.024)	1.006 (0.988, 1.026)	1.012 (0.994, 1.031)	1.013 (0.995, 1.031)
	NO_2_	10 µg/m^3^	1.026 (1.014, 1.039)	1.022 (1.008, 1.036)	-	0.993 (0.971, 1.015)	1.008 (0.993, 1.023)	1.004 (0.991, 1.017)	1.001 (0.988, 1.014)
	BC	0.5 × 10^–5^/m	1.036 (1.022, 1.051)	1.034 (1.019, 1.049)	1.043 (1.017, 1.070)	-	1.019 (1.002, 1.037)	1.014 (0.999, 1.029)	1.010 (0.995, 1.025)
	O_3_ warm	10 µg/m^3^	0.958 (0.946, 0.970)	0.960 (0.947, 0.974)	0.963 (0.948, 0.979)	0.969 (0.954, 0.985)	-	0.974 (0.961, 0.987)	0.975 (0.962, 0.988)
	PM_2.5_ Cu	5 ng/m^3^	1.048 (1.027, 1.069)	1.043 (1.021, 1.065)	1.042 (1.009, 1.076)	1.015 (0.979, 1.052)	1.023 (0.998,1.050)	1.023 (1.002, 1.044)	1.017 (0.996, 1.039)
	PM_2.5_ Fe	100 ng/m^3^	1.045 (1.023, 1.067)	1.038 (1.016, 1.061)	1.023 (0.987, 1.059)	0.981 (0.940, 1.023)	1.016 (0.990,1.043)	1.012 (0.990, 1.035)	1.005 (0.983, 1.028)
	PM_2.5_ S	200 ng/m^3^	1.018 (0.997, 1.040)	1.001 (0.976, 1.027)	0.994 (0.969, 1.020)	0.988 (0.965, 1.011)	0.983 (0.958,1.008)	0.998 (0.977, 1.020)	0.997 (0.975, 1.019)
	PM_2.5_ Zn	10 ng/m^3^	1.002 (0.997, 1.006)	1.000 (0.995, 1.006)	1.000 (0.995, 1.005)	0.999 (0.993, 1.004)	1.001 (0.995,1.006)	1.001 (0.996, 1.006)	1.001 (0.996, 1.006)
	Total Noise	10 dB Lden	1.041 (1.035, 1.047)	1.040 (1.034, 1.046)	1.040 (1.033, 1.047)	1.038 (1.031, 1.045)	1.037 (1.031, 1.044)	-	-
	Road traffic Noise	10 dB Lden	1.043 (1.037, 1.049)	1.042 (1.036, 1.048)	1.043 (1.036, 1.049)	1.041 (1.034, 1.047)	1.041 (1.034, 1.047)	-	-
Respiratory mortality	PM_2.5_	5 µg/m^3^	0.981 (0.953, 1.010)	-	0.933 0.901, 0.966)	0.925 (0.894, 0.956)	0.949 (0.917, 0.982)	0.963 (0.934, 0.992)	0.964 (0.935, 0.993)
	NO_2_	10 µg/m^3^	1.051 (1.031, 1.072)	1.079 (1.053, 1.105)	-	0.995 (0.956, 1.037)	1.036 (1.012, 1.061)	1.024 (1.003, 1.046)	1.020 (0.998, 1.042)
	BC	0.5 × 10^–5^/m	1.067 (1.043, 1.090)	1.101 (1.073, 1.130)	1.071 (1.024, 1.121)	-	1.055 (1.025, 1.085)	1.039 (1.016, 1.064)	1.034 (1.010, 1.059)
	O_3_ warm	10 µg/m^3^	0.947 (0.924, 0.971)	0.930 (0.905, 0.956)	0.968 (0.941, 0.997)	0.978 (0.948, 1.010)	-	0.969 (0.945, 0.994)	0.970 (0.946, 0.995)
	PM_2.5_ Cu	5 ng/m^3^	1.056 (1.021, 1.091)	1.075 (1.037, 1.113)	0.981 (0.928, 1.038)	0.926 (0.873, 0.983)	1.024 (0.985,1.065)	1.022 (0.988, 1.057)	1.015 (0.981, 1.05)
	PM_2.5_ Fe	100 ng/m^3^	1.092 (1.053, 1.133)	1.113 (1.070, 1.158)	1.058 (0.990, 1.129)	1.000 (0.927, 1.079)	1.068 (1.023,1.114)	1.052 (1.013, 1.092)	1.043 (1.004, 1.084)
	PM_2.5_ S	200 ng/m^3^	1.009 (0.974, 1.045)	1.035 (0.992, 1.079)	0.951 (0.909, 0.994)	0.944 (0.904, 0.985)	0.964 (0.926,1.004)	0.982 (0.948, 1.018)	0.980 (0.946, 1.015)
	PM_2.5_ Zn	10 ng/m^3^	0.999 (0.989, 1.009)	1.000 (0.990, 1.010)	0.996 (0.986, 1.006)	0.994 (0.984, 1.005)	0.998 (0.988,1.007)	0.998 (0.988, 1.008)	0.998 (0.988, 1.008)
	Total Noise	10 dB Lden	1.056 (1.043, 1.069)	1.059 (1.045, 1.072)	1.050 (1.037, 1.064)	1.048 (1.034, 1.061)	1.051 (1.038, 1.065)	-	-
	Road traffic Noise	10 dB Lden	1.058 (1.045, 1.071)	1.061 (1.047, 1.074)	1.053 (1.039, 1.067)	1.050 (1.036, 1.064)	1.054 (1.04, 1.067)	-	-

Model 3 (main model) adjusted for age, sex, individual level variables (education level, occupational status, marital status, origin, mother tongue), and area-level SES variables (composite score, unemployment rate, low education rate, high education rate). Additionally adjusted for noted exposure

“Total noise” refers to total transportation noise, i.e. the energetic sum of road traffic, railway and aircraft noise

Due to the high correlation between the exposures, the following two-pollutant models are difficult to interpret: between BC and NO_2_; Cu and NO_2_; Cu and BC; Fe and NO_2_; Fe and BC

In single exposure models, most exposures were also associated with CVD mortality (all except S and Zn). Similar to natural mortality, the associations were robust to adjustment for PM_2.5_; however, PM_2.5_ reduced somewhat after adjusting for most co-exposures. Adding noise to the models also reduced the associations to borderline significance for most pollutants, while noise was robust to adjustment for air pollution (Table 2).

For respiratory mortality, again most exposures showed associations in single exposure models (all except PM_2.5_, S and Zn). For this outcome only, the inverse association for O_3_ was reduced to unity when BC was included in the model. A robust association between noise and respiratory mortality was also found that persisted on co-exposure adjustment (i.e. 1.056 [1.043, 1.069] per 10 dB Lden total transportation noise vs. 1.048 [1.034, 1.061] per 10 dB Lden total transportation noise with BC adjustment) (Table 2).

Overall, the high correlations between NO_2_ and BC, as well as between NO_2_ / BC and Cu / Fe, made the two exposure models including these combinations difficult to interpret.

### Subset analysis on single exposure models

In subset analysis (Table 3), associations reported in the main Models 3 persisted below 20 µg/m^3^ PM_2.5_ for both natural (1.038 [1.026, 1.049] per 5 µg/m^3^) and CVD (1.039 [1.020, 1.058] per 5 µg/m^3^) mortality, however with virtually the entire study population (98.5%) residing in such areas. The point estimate was robust, indicative of associations down to the lowest investigated subset of below 12 µg/m^3^ PM_2.5_. For NO_2_, the results differed somewhat by outcome. For natural mortality, the association persisted down to the lowest subset of < 20 µg/m^3^, that included 31.8% of the study population (1.018 [1.000, 1.036] per 10 µg/m^3^). The associations for CVD and respiratory mortality remained < 30 µg/m^3^ (1.038 [1.022, 1.053] per 10 µg/m^3^ and 1.023 [0.993, 1.053] per 10 µg/m^3^, respectively). BC associations persisted within the lowest subset of < 1.5 10^–5^/m for both natural and CVD mortality, and to < 2 10^–5^/m for respiratory with hazard ratios of: 1.039 (1.013, 1.066), 1.051 (1.011, 1.093) and 1.039 (1.003, 1.076) per 0.5 × 10–5/m BC, respectively. Given the limited spatial variability in Switzerland in the higher range, the subset analysis for O_3_ warm was not informative compared to the full model. Finally, for total transportation noise, the natural mortality association remained down to < 53 dB with 1.012 (1.002, 1.023) per 10 dB Lden including only 37.3% of the study population, while CVD and respiratory associations clearly persisted < 60 dB Lden (1.028 [1.018,1.038] and 1.030 [1.007, 1.054] per 10 dB Lden, respectively).

**Table 3:** Subset analysis: hazard ratios (95% confidence intervals) for associations with mortality by cause, single exposure models (Model 3)

**Exposure**	**subset**	**N**	**%**	**Natural cause mortality**	**CVD mortality**	**Respiratory mortality**
PM_2.5_	Full	4,188,175	100.0	1.026 (1.015, 1.038)	1.026 (1.008, 1.044)	0.981 (0.953, 1.010)
	< 25	4,184,842	99.9	1.027 (1.017, 1.038)	1.028 (1.011, 1.046)	0.980 (0.951, 1.009)
	< 20	4,127,077	98.5	1.038 (1.026, 1.049)	1.039 (1.020, 1.058)	0.968 (0.933, 1.003)
	< 15	1,128,701	26.9	1.012 (0.992, 1.033)	1.016 (0.985, 1.049)	0.886 (0.829, 0.946)
	< 12	265,253	6.3	1.024 (0.983, 1.067)	1.051 (0.986, 1.120)	0.788 (0.690, 0.899)
NO_2_	Full	4,188,175	100.0	1.050 (1.041, 1.059)	1.026 (1.014, 1.039)	1.051 (1.031, 1.072)
	< 40	4,087,413	97.6	1.057 (1.049, 1.066)	1.034 (1.021, 1.047)	1.044 (1.020, 1.069)
	< 30	3,406,891	81.3	1.059 (1.049, 1.068)	1.038 (1.022, 1.053)	1.023 (0.993, 1.053)
	< 20	1,331,208	31.8	1.018 (1.000, 1.036)	0.996 (0.968, 1.024)	0.945 (0.889, 1.004)
BC	Full	4,188,175	100.0	1.057 (1.048, 1.067)	1.036 (1.022, 1.051)	1.067 (1.043, 1.090)
	< 3	4,177,681	99.7	1.060 (1.051, 1.070)	1.039 (1.024, 1.053)	1.068 (1.043, 1.093)
	< 2.5	4,094,565	97.8	1.066 (1.055, 1.076)	1.045 (1.029, 1.061)	1.064 (1.036, 1.092)
	< 2	3,494,997	83.4	1.074 (1.062, 1.086)	1.054 (1.034, 1.075)	1.039 (1.003, 1.076)
	< 1.5	1,471,268	35.1	1.039 (1.013, 1.066)	1.051 (1.011, 1.093)	0.945 (0.867, 1.030)
O_3_ warm	Full	4,188,175	100.0	0.946 (0.939, 0.954)	0.958 (0.946, 0.970)	0.947 (0.924, 0.971)
	< 120	4,188,175	100.0	0.946 (0.939, 0.954)	0.958 (0.946, 0.970)	0.947 (0.924, 0.971)
	< 100	3,411,297	81.5	0.933 (0.921, 0.945)	0.963 (0.945, 0.982)	0.896 (0.867, 0.926)
Total noise	Full	4,188,175	100.0	1.045 (1.040, 1.049)	1.041 (1.035, 1.047)	1.056 (1.043, 1.069)
	< 60	2,892,575	69.1	1.031 (1.025, 1.038)	1.028 (1.018, 1.038)	1.030 (1.007, 1.054)
	< 55	1,983,482	47.4	1.018 (1.009, 1.027)	1.008 (0.993, 1.022)	0.984 (0.953, 1.015)
	< 53	1,562,118	37.3	1.012 (1.002, 1.023)	0.998 (0.981, 1.015)	0.964 (0.929, 1.000)

Exposure increments: PM_2.5_ per 5 µg/m^3^, NO_2_ per 10 µg/m^3^, BC per 0.5 × 10^–5^/m, O_3_ warm per 10 µg/m^3^, Total (transportation) noise per 10 dB

Model 3 (main model) adjusted for age, sex, individual level variables (education level, occupational status, marital status, origin, mother tongue), and area-level SES variables (composite score, unemployment rate, low education rate, high education rate)

### Additional analyses on two exposure models

Regarding potential effect modification by sex, the association for total transportation noise (adjusted for PM_2.5_) was stronger in males compared to females. Indications of stronger effects in males compared to females were also found for NO_2_ and BC, but not PM_2.5_ or O_3_ warm in models that were mutually adjusted for noise (Table S6). Separately, stronger associations were found in non-movers compared to the full cohort again for NO_2_ and BC (adjusted for noise) and for total transportation noise (adjusted for PM_2.5_) (Table S7).

## Discussion

### Main findings

Single exposure models showed almost all air pollutants and noise exposures were positively associated with natural, cardiovascular and respiratory mortality outcomes. Many associations persisted at or below guideline limits for air pollution, as well as for natural mortality in relation to noise. Most associations were robust to adjustment for PM_2.5_ in two exposure models, however the opposite was not true. Specifically, associations for NO_2_, BC and O_3_ warm and both natural and respiratory mortality, as well as for BC and O_3_ warm for CVD mortality, largely remained after adjustment for co-exposures including transportation noise (total or road traffic noise only). Transportation noise was universally robust to adjustment for air pollution. As an outcome not considered in the WHO Environmental Noise Guidelines [16], the finding of an association between noise and respiratory mortality, independent of air pollution, was particularly novel but should be interpreted with caution given the lack of data on individual health behaviours.

The influence of co-exposure adjustment on the PM_2.5_ component associations with natural and CVD mortality was quite consistent, though it is important to acknowledge the high correlations when interpreting these findings. The single exposure associations for Cu and Fe did not change after adjustment for PM_2.5,_ while they attenuated after NO_2_ or BC adjustment. The attenuation was stronger for BC, which represents incomplete combustion. Further, the observation of BC being the more influential pollutant than PM_2.5_ supports the notion that the local air pollution mixture – and traffic related air pollution – may be more important than the broader regional mixture in Switzerland.

Another interesting finding, and in line with Vodonos et al. [36], is that the associations of the air pollutants almost universally strengthened with increasing covariate adjustment. This applied not only to the pollutants that showed increased risk, but also to the inverse O_3_ warm association. The exception was Zn that remained stable. The expectation in environmental epidemiology is that the effect estimates typically attenuate with better covariate adjustment. Here, however, better adjustment achieved by including several measures of area-level SEP at two spatial scales led to stronger HR. In Switzerland, built-up areas with higher levels of traffic related pollution are also those with typically higher socio-economic position thus explaining the negative confounding. This finding also challenges the idea that administrative cohorts over-estimate associations due to insufficient adjustment, though indeed lifestyle factors are not available in the SNC to more directly contest this assumption (see Strengths & limitations below).

### Comparison to previous literature on air pollution

The SNC was one of the administrative cohorts included in ELAPSE. Compared to the estimates from the meta-analysis including all cohorts, the Swiss effect estimates for natural mortality were slightly weaker for PM_2.5_ (1.026 [1.015, 1.038] Swiss vs. 1.053 [1.021, 1.085] combined per 5 µg/m^3^), slightly stronger for BC (1.057 [1.048, 1.067] vs. 1.039 [1.018, 1.059] per 0.5 × 10^–5^/m), and comparable for NO_2_ (1.050 [1.041, 1.059] vs. 1.044 [1.019, 1.069] per 10 µg/m^3^), and O_3_ warm (0.946 [0.939, 0.954] vs. 0.953 [0.929, 0.979] per 10 µg/m^3^) [22]. The associations reported in the pooled analysis of the eight detailed cohorts included in the other part of ELAPSE were stronger than in Switzerland [21].

Contrary to findings from North America [37–39], the inverse association for O_3_ warm was unexpected. Given that ozone is typically reduced by primary tail pipe emissions, one would expect the inverse association to disappear after adjusting for traffic pollutants. This was the behaviour observed in the meta-analysis of the seven administrative cohorts in ELAPSE; the single exposure O_3_ associations were inverse, though after co-pollutant adjustment the associations largely disappeared [22]. While HRs for Switzerland were marginally closer to unity after adjustment for co-exposures, the inverse relationship with ozone remained in two exposure models. We have no clear explanation for the robustness of the negative association in the Swiss cohort. This could relate to the moderately high negative correlations with the other exposures, perhaps made higher than previous studies given the 100 × 100 m spatial resolution. However, additional analyses in ELAPSE using O_3_ modelled at a coarser resolution likewise produced inverse associations for Switzerland [40].

The subset analysis for Switzerland revealed associations at lower levels of exposures for all air pollution-outcome pairs except O_3_ warm that had little exposure contrast. Generally studies from other regions have shown that the air pollution association with mortality persists in the low exposure range. These low-level cohort studies have tended to focus on PM_2.5_ exposure. The Canadian studies, specifically three waves of the Canadian Census Health and Environment Cohort (CanCHEC) and the Canadian Community Health Survey (CCHS), observed supralinear associations between PM_2.5_ and mortality starting at very low levels [4, 9]. In the US Medicare cohort, associations were also found with PM_2.5_, NO_2_, and summer O_3_ below the air quality standard levels [41]. Similar to these findings for Switzerland, the effect of PM_2.5_ on mortality in the Canadian studies attenuated when NO_2_ was considered [4, 9]. Findings in the Sydney, Australia ’45 and Up Study also suggested both PM_2.5_ and NO_2_ were related to premature mortality [7]. None of these studies, except for ELAPSE, considered co-adjustment with transportation noise.

### Role of transportation noise

It is not yet common practice to adjust for noise in the wider literature on health effects of traffic related air pollution. The several studies on mortality, however, are available to shed more light on the issue of potential confounding in different study areas and populations in Europe. The Danish Diet, Cancer and Health Cohort reported indicative associations of CVD and all-cause mortality with several air pollutants, after substantial attenuation with noise adjustment [42]. Others, however, found little effect of noise adjustment [43–46]. Within ELAPSE, adjustment for traffic noise hardly changed the association between air pollutants and CVD mortality for most of the included administrative cohorts; associations slightly strengthened for the Dutch cohort and slightly attenuated for the others [22]. The impact of noise adjustment across these cohorts in ELAPSE likely relates to the urban structure and subsequent degree of correlations. Also, unlike air pollution where exposures were from harmonized Europe-wide models, differences in the quality, specification or resolution of the country-specific noise models may play a role. High quality country-specific noise models are expected to better capture small-scale variation in exposures [47]. In Switzerland, the correlations between air pollution and noise exposures were not high. Further the exposure models were all comparably high resolution which should minimize the possibility of effect transfer, i.e. when the effect from the less well measured exposure is transferred to (or mopped up by) the better measured one [42, 48, 49].

Previously using the same data for Switzerland, Vienneau et al. [47] demonstrated the sensitivity of noise exposure assessment illustrating that use of fine scale noise maps rather than estimates directly at the household façade underestimates the associations. Several cohorts in ELAPSE, in addition to Switzerland, used state-of-the-art emissions and propagation models (described in [22]) which is the gold standard for noise exposure modelling. Detailed in Karipidis et al. [31], the Swiss noise exposure data were available at the façade of the residential location including floor of residence. The models have been shown to perform well in a validation study using measurements at the window (agreement between measured and modelled: mean + 0.5 dB(A), standard deviation 4.0 dB(A)) [50]. Previous Swiss studies on exposure to transportation noise sources and CVD mortality found that associations were robust to air pollution adjustment [12]. This current study, with more detailed air pollution modelling and a broader range of air pollutants strengthens the evidence that transportation noise is an independent risk factor for natural, CVD and respiratory mortality. It should be noted, however, that the subset analysis suggests the air pollution associations are largely linear while noise may have a threshold (i.e., for CVD and respiratory mortality). Thus, the impact of noise adjustment on the air pollution associations for some outcomes may differ between low and high exposure groups. Another concern with noise models, similar to models for air pollution, is that they reflect ambient conditions rather than indoor exposures which may contribute to exposure misclassification. Foraster et al. [51] showed that modelling indoor noise at the bedroom substantially reduced correlations with air pollution and led to more consistent associations with blood pressure in a cross-sectional study in Gerona, Spain. Similar correction factors have been investigated by Locher et al. [52] in Switzerland, though lack of national data on noise attenuation factors limits their application in the SNC. In this study, however, any exposure misclassification is expected to be non-differential.

To date, the strongest evidence for health effects of transportation noise relates to CVD outcomes [13, 15, 53–55]. In addition to CVD mortality, the findings reported here also show that transportation noise was associated with natural and respiratory mortality. The few similar studies also concur with an association for natural mortality, though are less consistent for respiratory mortality. In the Danish Nurse Cohort (DNC), Liu et al. [17] reported a HR of 1.15 (1.06, 1.25) per 10 dB Lden road traffic noise in association with chronic obstructive pulmonary disease. In the same cohort, road traffic noise was associated with all-cause mortality (1.09 [1.03, 1.15] per 10 dB 23-year mean Lden), and suggestive for respiratory mortality (1.16 [0.97, 1.40]) [11]. In both DNC studies, the associations similarly persisted after adjusting for air pollution. The Danish Diet, Cancer and Health Cohort study also found associations with road traffic noise and both CVD (1.13 [1.06, 1.19] 10-year mean Lden at the most exposed façade) and all-cause mortality (1.08 [1.05, 1.11], but not with respiratory mortality (1.02 [0.96, 1.09]) [14]. In the Swiss SAPALDIA study, transportation noise was found to be associated with exacerbation of asthma symptoms in asthmatics, but not incidence of asthma [33]. Noise has further been suggested as a risk factor for diabetes and neurodegenerative diseases [56–58]. Hence, there is an increased rationale for assessing mortality from natural causes in relation to noise, in addition to the more traditional cardiovascular causes. Interestingly, associations with noise below 53 dB (the WHO guideline value for road traffic noise) remained significant for natural mortality but not CVD mortality. Note this was based on total transportation noise, though given the near identical associations for road traffic noise we expect the same finding. That the association for natural mortality persists indicates a cause other than CVD is strongly related to noise at low levels.

### Biological mechanisms

The underlying mechanisms by which long term air pollution exposure is related to CVD is thought to be oxidative stress and systematic inflammation. The same mechanisms likely play a role in respiratory disease [59, 60]. For noise, research into mechanisms has focused on elucidating the cardio-metabolic pathways, stemming from direct (auditory) sleep disturbance and indirect (non-auditory) disturbances such as annoyance. Noise can trigger a physiological stress response through activation of the Hypothalamic–Pituitary–Adrenal axis. This includes the releases of stress hormones, leading to increased blood pressure, vascular dysfunction, inflammation and oxidative stress [13, 61–63]. Being in a stressed state may also disrupt night-time recovery of the immune system, contributing to inflammation and oxidative stress also in the respiratory tract [63].

There is growing evidence that air pollution accelerates aging, and that multiple organs can be affected [60, 64]. In addition to reduced longevity, aging-related outcomes that have been associated with air pollution range from reduced lung function, increased chronic disease, frailty, to cognitive decline [65–69]. Sleep, which can be disrupted by noise, also changes with aging, with poor sleep linked to many adverse health outcomes [70]. Regarding the cause-specific mortalities investigated here, whether air pollution or noise first affects an individual’s heart or lungs may relate to comorbidities. Self-selection may also be important as suggested in the subset analysis where the effects generally attenuated in those living in less polluted areas. This was particularly noticeable for respiratory mortality, where an inverse association for PM_2.5_ was found in the lowest subsets. It is plausible that those with severe respiratory disease intentionally move to less polluted areas.

### Strengths & limitations

The strengths of this analysis included the large study population followed for up to 14 years. The cohort had near complete coverage given that the census in Switzerland is compulsory, minimizing selection bias. Both individual and area-level covariates at baseline could be derived from the same source; the 04 December 2000 census. High spatial resolution air pollution and noise exposures were linked to this population at their place of residence.

Given the importance of sleep as one mechanism for noise effects, it may be reasonable to specifically investigate night-time noise exposure in addition to Lden that covers the full 24-h day with an added penalty for evening and night hours. The correlations between Lden and Leq,night in the study population, however, are near identical (*r* = 0.99 total transportation noise, and 1.00 for road traffic noise). Further, Lden is the main metric used in studies on chronic health effects, including in the WHO noise guidelines (Leq,night is only used in relation to sleep disturbance) [16]. Individual data on behavioural risk factors were not available in the SNC, thus direct adjustment for such factors could not be performed. However, including individual behaviours has been shown to only modestly attenuate the mortality associations for PM_2.5_ in some studies that could investigate this in detail [4, 71]. Within ELAPSE, cohort-specific sensitivity analyses – including for the SNC – showed natural mortality associations were robust to indirect adjustment for smoking and body-mass index (Table S13 in Stafoggia et al. [22]). Whether this also holds for other mortality outcomes or exposures requires further study. Another possible limitation is that the air pollution exposures related to year 2010 [25, 26] which may introduce some exposure misclassification. For natural mortality, additional sensitivity analysis within ELAPSE included back extrapolating these 2010 exposures to the baseline year 2000 (Table S14 in Stafoggia et al. [22]). Accounting for residential history (with 34% participants known to have moved during follow up, the results for Switzerland based on back extrapolated exposures were found to be robust. Back extrapolation, however, was applied in each ELAPSE study area at NUTS-2 regions (Nomenclature of territorial units for statistics, harmonised hierarchical system for Europe, which for Switzerland equals national scale making this a temporal correction only. It is also possible that the true association is diluted due to exposure misclassification from assigning exposure only at baseline. While address history is available in the SNC, as indicated above, the intervals are inconsistent. It is collected via the census at 2000 then annually from 2010 onward. For the gap between 2000 and 2010, we rely on the change in geocode/building ID plus a census question related to community of residence at 2006 to identify movers and broadly assign the timing of a move (i.e. before vs after 2006). Any information about moving for those who die between 2000 and 2010, however, is lost. The sensitivity analysis restricting to non-movers indeed showed stronger associations for NO_2_, BC and total transportation noise compared to the full cohort. This suggests exposure misclassificaton may be particularly relevant for the more local/traffic-related exposures. Finally, investigating mortality endpoints does not enable differentiating effects on disease etiology from the effects on disease progression.

### Policy implications

While the current air quality and environmental noise guidelines focus on regulating single exposures that are pivotal for health protection, more attention is needed regarding the complex multipollutant mixture [72]. This includes gaining insight into the area-specific composition of PM as well as attempting to disentangle associations with noise. As demonstrated in this study, however, the PM components were highly correlated with other exposures lending to results that could not be easily interpreted. The 2021 WHO AQG have set limit values for PM_2.5_, NO_2_ and O_3_ in the peak warm season which, for the annual averaging period, are 5 µg/m^3^, 10 µg/m^3^ and 60 µg/m^3^ [3]. These limits are notably lower than the majority of exposures in Switzerland according to this study. The AQG also include good practice statements, aimed to draw attention to other pollutants conveying risk but with less supporting evidence to develop a limit value such as BC. Though at present there is also no Swiss limit value for BC, this pollutant is of particular concern. As emphasized in a 2013 PM report by the Federal Commission for Air Hygiene (FCAH), BC is considered carcinogenic. Thus, the concentrations should be as low as possible to limit the consequences to not more than 1 case per 1 million [73], that approximately corresponds to an annual mean concentration of 0.1 µg/m^3^ soot (elemental carbon). FACH called for a reduction strategy to reach 20% of the 2013 levels within 10 years, given still far higher levels of 2–3 µg/m^3^ observed in many urban locations. ELAPSE and this analysis in the SNC underscore the call for continued and rigorous reductions of BC to protect public health. It also strongly suggests that additional outcomes beyond CVD should be considered when evaluating the burden of disease from transportation noise.

### Conclusion

In this study using a large administrative cohort, long-term exposure to air pollution and transportation noise were associated with mortality in Switzerland. Traffic-related air pollutants were more strongly associated with natural, CVD and respiratory mortality than PM_2.5_ mass and components. Noise was not only associated with CVD, but also with respiratory and natural cause mortality. Associations with noise remained for natural cause mortality below the WHO guideline value for road traffic noise of 53 dB Lden. Considering co-exposures revealed the importance of local traffic-related pollutants such as NO_2_ and BC as well as transportation noise in relation to mortality.

## Supplementary Information

**Additional file 1: Table S1.** Study population selection. **Table S2.** Exposure distributions. **Table S3.** Spearman correlations between exposures (top), and between exposures and neighbourhood socio-economic position (SEP) score (bottom). **Table S4.** Mean exposure by quintiles of neighbourhood socio-economic position. **Table S5.** Hazard ratios (95% confidence intervals) for single exposure associations between air pollution and noise exposures and mortality by cause, with increasing level of covariate adjustment in single exposure models. **Table S6.** Effect modification by sex, hazard ratios (95% confidence intervals) in two exposure models for associations with natural cause mortality (Model 3). **Table S7.** Non-mover analysis, hazard ratios (95% confidence intervals) in two exposure models for associations with natural cause mortality (Model 3).

## Data Availability

The SNC data cannot be shared by the authors. The Federal Statistical Office is responsible for the SNC data; information regarding data requests is available here: https://www.bfs.admin.ch/bfs/en/home/statistics/population/surveys/snc.html.
