# Supplementary material for: Association between exposure to multiple air pollutants, transportation noise and cause-specific mortality in adults in Switzerland
Source: Environ Health. 2023 Mar 27;22:29. doi: 10.1186/s12940-023-00983-y (PMC10041702; doi:10.1186/s12940-023-00983-y)
Supplement: Supplementary file 1 — Additional file 1: Table S1. Study population selection. Table S2. Exposure distributions. Table S3. Spearman correlations between exposures (top), and between exposures and neighbourhood socio-economic position (SEP) score (bottom). Table S4. Mean exposure by quintiles of neighbourhood socio-economic position. Table S5. Hazard ratios (95% confidence intervals) for single exposure associations between air pollution and noise exposures and mortality by cause, with increasing level of covariate adjustment in single exposure models. Table S6. Effect modification by sex, hazard ratios (95% confidence intervals) in two exposure models for associations with natural cause mortality (Model 3). Table S7. Non-mover analysis, hazard ratios (95% confidence intervals) in two exposure models for associations with natural cause mortality (Model 3). [file 12940_2023_983_MOESM1_ESM.docx]

Supplement

# **Association between exposure to multiple fine scale air pollutants, transportation noise and cause-specific mortality in adults in Switzerland**

Danielle Vienneau^1,2^, Massimo Stafoggia^3,4^, Sophia Rodopoulou^5^, Jie Chen^6^, Richard W Atkinson^7^, Mariska Bauwelinck^8^, Jochem O Klompmaker^9^, Bente Oftedal^10^, Zorana J Andersen^11^, Nicole A H Janssen^9^, Rina So^11^, Youn-Hee Lim^11^, Benjamin Flückiger^1,2^, Regina Ducret-Stich^1,2^, Martin Röösli^1,2^, Nicole Probst-Hensch^1,2^, Nino Künzli^1,2^, Maciek Strak^9^, Evangelia Samoli^5^, Kees de Hoogh^1,2^, Bert Brunekreef^6^, Gerard Hoek^6^

Table S1. Study population selection

| **Description** | **N** | **% total** | **% excluded** | **Notes** |
| --- | --- | --- | --- | --- |
| Full SNC | 12194229 |  |  |  |
| Baseline (in cohort 04.12.2000) | 7280246 |  | 0 | Full population (original sample) |
| Keep adults 30+ | 4689212 | 100 | 35.6 | Adult population (thus 35.6% dropped from full population baseline) |
| Exclude invalid xy coordinates & household ID | 4573377 | 97.5 | 2.5 |  |
| Exclude housing type = hotels, hospitals, old persons homes | 4293521 | 91.6 | 6.1 | N participants total |
| Exclude education = child or unknown | 4193954 | 89.4 | 2.3 |  |
| Exclude missing SSEP index | 4188175 | 89.3 | 0.1 | N participants with complete data (included in analysis) |

Table S2 Exposure distributions

| **Exposure** | **N** | **Mean** | **SD** | **Min** | **p5** | **p25** | **Median** | **p75** | **p95** | **Max** | **IQR** |
| --- | --- | --- | --- | --- | --- | --- | --- | --- | --- | --- | --- |
| PM_2.5_ (µg/m^3^) | 4188175 | 15.9 | 2.4 | 0.6 | 11.6 | 14.9 | 16.3 | 17.4 | 19.0 | 28.0 | 2.6 |
| NO_2_ (µg/m^3^) | 4188175 | 23.7 | 7.4 | 1.8 | 12.5 | 18.8 | 23.0 | 28.0 | 37.1 | 82.9 | 9.2 |
| BC (10^-5^/m) | 4188175 | 1.67 | 0.35 | 0.84 | 1.21 | 1.43 | 1.61 | 1.86 | 2.32 | 4.87 | 0.43 |
| O3 warm (µg/m^3^) | 4188175 | 94.8 | 5.9 | 56.2 | 86.2 | 90.9 | 94.0 | 98.1 | 106.0 | 116.0 | 7.2 |
| PM_2.5_ Cu (ng/m^3^) | 4188175 | 5.2 | 2.7 | 0.0 | 1.1 | 3.5 | 4.8 | 6.7 | 10.1 | 24.7 | 3.2 |
| PM_2.5_ Fe (ng/m^3^) | 4188175 | 108.4 | 46.9 | 10.6 | 47.6 | 75.9 | 97.9 | 133.9 | 198.8 | 472.2 | 58.0 |
| PM_2.5_ S (ng/m^3^) | 4188175 | 646.6 | 85.0 | 302.0 | 469.1 | 612.3 | 655.0 | 693.9 | 774.1 | 1113.8 | 81.6 |
| PM_2.5_ Zn (ng/m^3^) | 4188175 | 20.8 | 18.5 | 0.0 | 3.3 | 11.7 | 16.1 | 24.7 | 47.3 | 145.4 | 13.0 |
| Total transportation noise (dB Lden) | 4188175 | 55.9 | 8.2 | 35.0 | 42.6 | 50.4 | 55.5 | 61.6 | 69.3 | 92.2 | 11.2 |
| Road traffic noise (dB Lden) | 4188175 | 54.2 | 8.1 | 35 | 40.8 | 48.7 | 53.8 | 60.0 | 67.9 | 85.4 | 11.3 |

Table S3 Spearman correlations between exposures (top), and between exposures and neighbourhood socio-economic position (SEP) score (bottom)

| **Exposure** | **PM_2.5_** | **NO_2_** | **BC** | **O_3_** | **PM_2.5_ Cu** | **PM_2.5_ Fe** | **PM_2.5_ S** | **PM_2.5_ Zn** | **Total noise** | **Road traffic noise** |
| --- | --- | --- | --- | --- | --- | --- | --- | --- | --- | --- |
|  |  |  |  | **warm** |  |  |  |  |  |  |
| PM_2.5_ | 1 |  |  |  |  |  |  |  |  |  |
| NO_2_ | 0.72 | 1 |  |  |  |  |  |  |  |  |
| BC | 0.74 | 0.91 | 1 |  |  |  |  |  |  |  |
| O_3_ warm | -0.65 | -0.68 | -0.68 | 1 |  |  |  |  |  |  |
| PM_2.5_ Cu | 0.68 | 0.88 | 0.90 | -0.65 | 1 |  |  |  |  |  |
| PM_2.5_ Fe | 0.67 | 0.91 | 0.92 | -0.66 | 0.97 | 1 |  |  |  |  |
| PM_2.5_ S | 0.66 | 0.70 | 0.69 | -0.68 | 0.67 | 0.70 | 1 |  |  |  |
| PM_2.5_ Zn | 0.62 | 0.70 | 0.73 | -0.52 | 0.75 | 0.77 | 0.65 | 1 |  |  |
| Total noise | 0.29 | 0.40 | 0.40 | -0.32 | 0.35 | 0.38 | 0.31 | 0.21 | 1 |  |
| Road traffic noise | 0.25 | 0.39 | 0.40 | -0.30 | 0.33 | 0.36 | 0.29 | 0.18 | 0.89 | 1 |
|  |  |  |  |  |  |  |  |  |  |  |
| SEP score | 0.32 | 0.30 | 0.32 | -0.28 | 0.39 | 0.36 | 0.32 | 0.30 | 0.02 | 0.01 |

“Total noise” refers to total transportation noise, i.e. the energetic sum of road traffic, railway and aircraft noise

Table S4 Mean exposure by quintiles of neighbourhood socio-economic position

| **SEP quintile** | **PM_2.5_**  (µg/m^3^) | **NO_2_**  (µg/m^3^) | **BC**  (10^-5^/m) | **O_3_ warm** (µg/m^3^) | **PM_2.5_ Cu**  (ng/m^3^) | **PM_2.5_ Fe**  (ng/m^3^) | **PM_2.5_ S**  (ng/m^3^) | **PM_2.5_ Zn**  (ng/m^3^) | **Total noise**  (dB Lden) |
| --- | --- | --- | --- | --- | --- | --- | --- | --- | --- |
| Full population | | | | | | | | | |
| 1 (low) | 14.7 | 19.9 | 1.5 | 98.2 | 3.6 | 83.6 | 595.3 | 16.9 | 55.1 |
| 2 | 15.8 | 23.4 | 1.6 | 95.2 | 4.9 | 103.4 | 642.5 | 20.4 | 56.2 |
| 3 | 16.1 | 25.0 | 1.7 | 93.5 | 5.6 | 117.5 | 655.2 | 22.5 | 56.3 |
| 4 | 16.3 | 24.0 | 1.7 | 94.2 | 5.4 | 111.5 | 658.9 | 20.6 | 55.9 |
| 5 (high) | 16.8 | 26.3 | 1.8 | 92.9 | 6.4 | 126.0 | 681.2 | 23.6 | 55.9 |
| Urban (n=1203677) | | | | | | | | | |
| 1 (low) | 16.1 | 25.2 | 1.7 | 95.3 | 5.3 | 110.7 | 641.4 | 24.4 | 57.8 |
| 2 | 17.6 | 31.5 | 2.1 | 90.3 | 8.0 | 160.7 | 697.5 | 28.4 | 59.2 |
| 3 | 16.4 | 28.3 | 1.9 | 91.9 | 6.7 | 142.1 | 668.4 | 32.7 | 58.4 |
| 4 | 17.1 | 28.1 | 1.9 | 91.8 | 7.1 | 143.6 | 671.4 | 27.8 | 57.2 |
| 5 (high) | 17.2 | 31.3 | 2.1 | 90.7 | 8.1 | 161.2 | 702.0 | 33.2 | 57.4 |
| Peri-urban (1915622) | | | | | | | | | |
| 1 (low) | 16.0 | 24.0 | 1.7 | 96.9 | 5.0 | 106.6 | 645.3 | 29.4 | 56.6 |
| 2 | 16.1 | 24.2 | 1.7 | 94.4 | 5.4 | 110.5 | 650.8 | 19.0 | 55.6 |
| 3 | 16.0 | 23.1 | 1.6 | 95.3 | 4.9 | 102.2 | 653.5 | 20.0 | 55.8 |
| 4 | 16.4 | 24.3 | 1.7 | 93.8 | 5.5 | 111.3 | 671.8 | 19.6 | 55.7 |
| 5 (high) | 16.8 | 23.9 | 1.7 | 93.9 | 5.7 | 110.7 | 671.1 | 18.8 | 55.2 |
| Rural (n=1068876) | | | | | | | | | |
| 1 (low) | 13.1 | 15.0 | 1.3 | 100.8 | 2.1 | 58.6 | 543.4 | 6.1 | 53.5 |
| 2 | 14.2 | 17.2 | 1.4 | 98.3 | 2.7 | 67.2 | 582.1 | 9.9 | 53.7 |
| 3 | 14.2 | 17.3 | 1.4 | 98.0 | 2.7 | 66.7 | 588.0 | 9.8 | 53.6 |
| 4 | 14.6 | 18.7 | 1.4 | 96.7 | 3.3 | 74.0 | 609.1 | 12.0 | 53.9 |
| 5 (high) | 15.5 | 19.4 | 1.4 | 95.3 | 3.7 | 77.9 | 637.7 | 13.5 | 53.8 |

Table S5 Hazard ratios (95% confidence intervals) for single exposure associations between air pollution and noise exposures and mortality by cause, with increasing level of covariate adjustment in single exposure models

| **Outcome** | **Exposure** | **Increment** | **Model 1** | **Model 2** | **Model 3** |
| --- | --- | --- | --- | --- | --- |
|  |  |  | HR (95% CI) | HR (95% CI) | HR (95% CI) |
| Natural cause mortality | PM_2.5_ | 5 µg/m^3^ | 0.979 (0.966, 0.993) | 1.007 (0.997, 1.018) | 1.026 (1.015, 1.038) |
|  | NO_2_ | 10 µg/m^3^ | 1.005 (0.993, 1.016) | 1.020 (1.012, 1.028) | 1.050 (1.041, 1.059) |
|  | BC | 0.5 x 10^-5^/m | 1.008 (0.996, 1.021) | 1.022 (1.014, 1.030) | 1.057 (1.048, 1.067) |
|  | O_3_ warm | 10 µg/m^3^ | 0.976 (0.964, 0.988) | 0.968 (0.960, 0.977) | 0.946 (0.939, 0.954) |
|  | PM_2.5_ Cu | 5 ng/m^3^ | 0.991 (0.975, 1.008) | 1.013 (1.001, 1.025) | 1.067 (1.054, 1.080) |
|  | PM_2.5_ Fe | 100 ng/m^3^ | 1.005 (0.985, 1.025) | 1.025 (1.011, 1.039) | 1.085 (1.070, 1.100) |
|  | PM_2.5_ S | 200 ng/m^3^ | 0.989 (0.971, 1.007) | 1.012 (0.999, 1.025) | 1.035 (1.020, 1.051) |
|  | PM_2.5_ Zn | 10 ng/m^3^ | 1.004 (1.001, 1.008) | 1.005 (1.003, 1.008) | 1.004 (1.002, 1.006) |
|  | Total noise | 10 dB Lden | 1.051 (1.045, 1.057) | 1.044 (1.039, 1.049) | 1.045 (1.040, 1.049) |
| CVD mortality | PM_2.5_ | 5 µg/m^3^ | 0.888 (0.867, 0.909) | 0.942 (0.927, 0.957) | 1.026 (1.008, 1.044) |
|  | NO_2_ | 10 µg/m^3^ | 0.916 (0.900, 0.933) | 0.954 (0.945, 0.964) | 1.026 (1.014, 1.039) |
|  | BC | 0.5 x 10^-5^/m | 0.918 (0.900, 0.937) | 0.953 (0.942, 0.964) | 1.036 (1.022, 1.051) |
|  | O_3_ warm | 10 µg/m^3^ | 1.042 (1.018, 1.066) | 1.025 (1.011, 1.040) | 0.958 (0.946, 0.970) |
|  | PM_2.5_ Cu | 5 ng/m^3^ | 0.876 (0.853, 0.899) | 0.923 (0.909, 0.937) | 1.048 (1.027, 1.069) |
|  | PM_2.5_ Fe | 100 ng/m^3^ | 0.869 (0.842, 0.897) | 0.917 (0.901, 0.933) | 1.045 (1.023, 1.067) |
|  | PM_2.5_ S | 200 ng/m^3^ | 0.907 (0.883, 0.930) | 0.935 (0.919, 0.952) | 1.018 (0.997, 1.040) |
|  | PM_2.5_ Zn | 10 ng/m^3^ | 0.987 (0.976, 0.997) | 0.990 (0.983, 0.998) | 1.002 (0.997, 1.006) |
|  | Total noise | 10 dB Lden | 1.017 (1.007, 1.027) | 1.025 (1.018, 1.031) | 1.041 (1.035, 1.047) |
| Respiratory mortality | PM_2.5_ | 5 µg/m^3^ | 0.931 (0.900, 0.962) | 0.943 (0.918, 0.970) | 0.981 (0.953, 1.010) |
|  | NO_2_ | 10 µg/m^3^ | 1.001 (0.980, 1.022) | 0.999 (0.983, 1.016) | 1.051 (1.031, 1.072) |
|  | BC | 0.5 x 10^-5^/m | 1.004 (0.982, 1.027) | 1.005 (0.987, 1.022) | 1.067 (1.043, 1.090) |
|  | O_3_ warm | 10 µg/m^3^ | 1.018 (0.992, 1.044) | 1.009 (0.986, 1.031) | 0.947 (0.924, 0.971) |
|  | PM_2.5_ Cu | 5 ng/m^3^ | 0.974 (0.943, 1.006) | 0.980 (0.957, 1.004) | 1.056 (1.021, 1.091) |
|  | PM_2.5_ Fe | 100 ng/m^3^ | 0.995 (0.962, 1.030) | 0.997 (0.971, 1.023) | 1.092 (1.053, 1.133) |
|  | PM_2.5_ S | 200 ng/m^3^ | 0.920 (0.886, 0.956) | 0.950 (0.920, 0.982) | 1.009 (0.974, 1.045) |
|  | PM_2.5_ Zn | 10 ng/m^3^ | 0.992 (0.981, 1.003) | 0.996 (0.987, 1.004) | 0.999 (0.989, 1.009) |
|  | Total noise | 10 dB Lden | 1.079 (1.064, 1.094) | 1.049 (1.036, 1.062) | 1.056 (1.043, 1.069) |

Model 1 adjusted for age, sex

Model 2 additionally adjusted for individual level variables (education level, occupational status, marital status, origin, mother tongue)

Model 3 (main model) additionally adjusted for area-level SES variables (composite score, unemployment rate, low education rate, high education rate)

“Total noise” refers to total transportation noise, i.e. the energetic sum of road traffic, railway and aircraft noise

|  |  |  |  |  |  |  |  |  |  |  |  |  |
| --- | --- | --- | --- | --- | --- | --- | --- | --- | --- | --- | --- | --- |

Table S6 Effect modification by sex, hazard ratios (95% confidence intervals) in two exposure models for associations with natural cause mortality (Model 3)

| **Outcome** | **Exposure** | **Adjusted for** | **Increment** | **Males** | **Females** |
| --- | --- | --- | --- | --- | --- |
|  |  |  |  | HR (95% CI) | HR (95% CI) |
| Natural cause mortality | PM_2.5_ | Total transportation noise | 5 µg/m^3^ | 1.016 (1.004, 1.029) | 1.008 (0.996, 1.021) |
|  | NO_2_ |  | 10 µg/m^3^ | 1.039 (1.029, 1.048) | 1.022 (1.012, 1.031) |
|  | BC |  | 0.5 x 10^-5^/m | 1.044 (1.034, 1.055) | 1.029 (1.019, 1.039) |
|  | O_3_ warm |  | 10 µg/m^3^ | 0.962 (0.953, 0.971) | 0.962 (0.952, 0.971) |
|  | Total noise | PM_2.5_ | 10 dB Lden | 1.059 (1.053, 1.064) | 1.029 (1.024, 1.035) |

Model 3 (main model) adjusted for age, individual level variables (education level, occupational status, marital status, origin, mother tongue), and area-level SES variables (composite score, unemployment rate, low education rate, high education rate). Additionally adjusted for noted exposure.

“Total noise” refers to total transportation noise, i.e. the energetic sum of road traffic, railway and aircraft noise

Table S7 Non-mover analysis, hazard ratios (95% confidence intervals) in two exposure models for associations with natural cause mortality (Model 3)

| **Outcome** | **Exposure** | **Adjusted for** | **Increment** | **Full Cohort**  (n=4188175) | **Non-movers**  (n=2747542) |
| --- | --- | --- | --- | --- | --- |
|  |  |  |  | HR (95% CI) | HR (95% CI) |
| Natural cause mortality | PM_2.5_ | Total transportation noise | 5 µg/m^3^ | 1.012 (1.001, 1.023) | 1.010 (0.999, 1.021) |
|  | NO_2_ |  | 10 µg/m^3^ | 1.029 (1.021, 1.038) | 1.039 (1.030, 1.048) |
|  | BC |  | 0.5 x 10^-5^/m | 1.035 (1.026, 1.044) | 1.042 (1.032, 1.052) |
|  | O_3_ warm |  | 10 µg/m^3^ | 0.962 (0.955, 0.970) | 0.976 (0.967, 0.985) |
|  | Total noise | PM_2.5_ | 10 dB Lden | 1.044 (1.039, 1.048) | 1.051 (1.046, 1.056) |

Model 3 (main model) adjusted for age, sex, individual level variables (education level, occupational status, marital status, origin, mother tongue), and area-level SES variables (composite score, unemployment rate, low education rate, high education rate). Additionally adjusted for noted exposure.

“Total noise” refers to total transportation noise, i.e. the energetic sum of road traffic, railway and aircraft noise
